# [Corrigendum] Targeting Sirt1 in a rat model of high-fat diet-induced non-alcoholic fatty liver disease: Comparison of Gegen Qinlian decoction and resveratrol

**DOI:** 10.3892/etm.2026.13184

**Published:** 2026-05-13

**Authors:** Yi Guo, Jun-Xiang Li, Tang-You Mao, Wei-Han Zhao, Li-Juan Liu, Yun-Liang Wang

Exp Ther Med 14:4279–4287, 2017; DOI: 10.3892/etm.2017.5076

Following the publication of the above paper, an interested reader drew to the Editor’s attention that, for the histological data shown in Figs. 3 and 4, panels A-D of these five-panel figures had previously appeared in a paper by the same research group that was published in the journal *Evidence-Based Complementary and Alternative Medicine*. Upon asking the authors for an explanation, they replied to explain that the previous publication should have been acknowledged as the source of these data, as they were essentially included in these figures for comparative purposes with the new data presented in panels E of the figures. Following a consultation with the Editorial Office, and given that these data had already been published, the authors have agreed to proceed with a corrigendum featuring data from one of the repeated experiments for panels A-D in this pair of figures, and the revised versions of Figs. 3 and 4 are therefore shown on the next page.

Note that the revisions made to these figures do not affect either the results or the conclusions reported in this paper. All the authors agree with the publication of this corrigendum, and are grateful to the Editor of *Experimental and Therapeutic Medicine* for granting them the opportunity to publish this; furthermore, they apologize to the readership for any inconvenience caused.

## Figures and Tables

**Figure 3:**
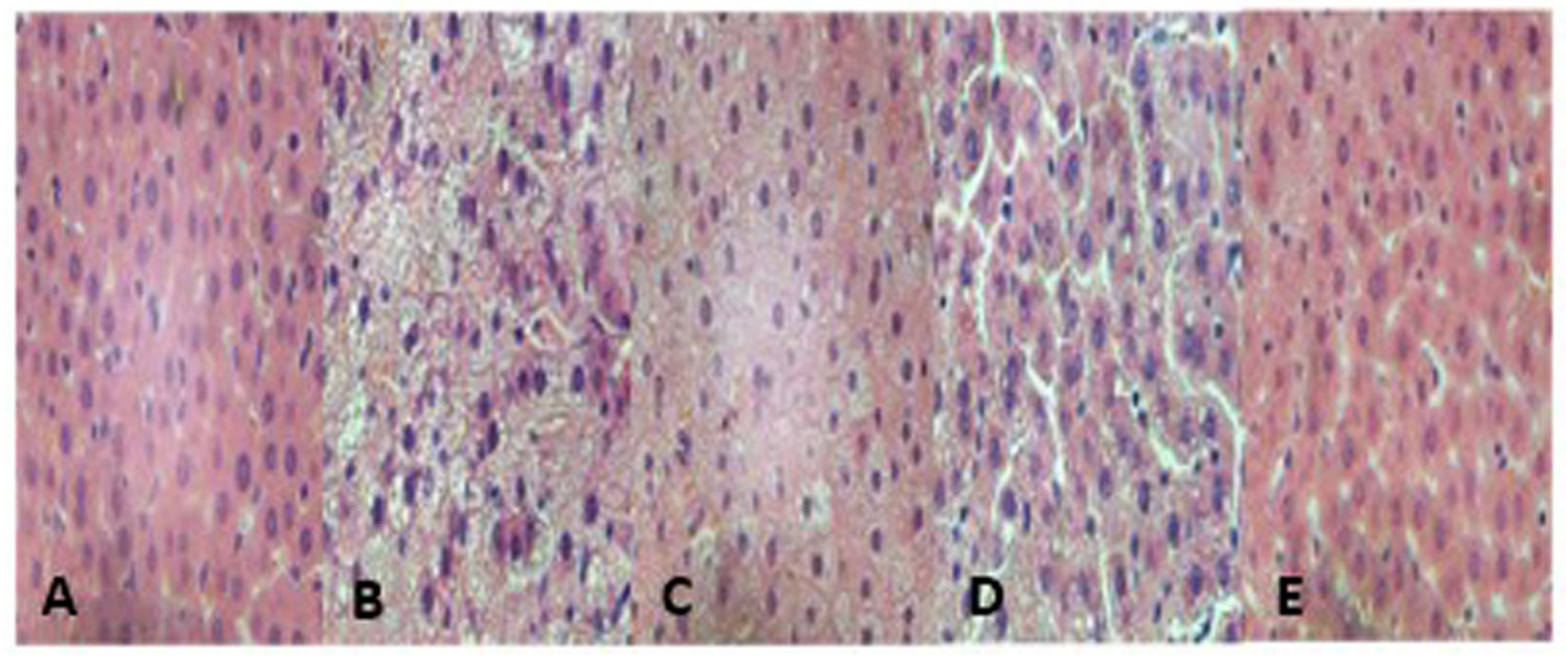
Results of paraffin section and haematoxylin & eosin staining *in vivo* (magnification, ×100). Images of liver tissues from (A) chow-fed, (B) high-fat diet model, (C) GGQLD-low dose, (D) GGQLD-high dose and (E) resveratrol-treated rats. GGQLD, Gegen Qinlian decoction.

**Figure 4:**
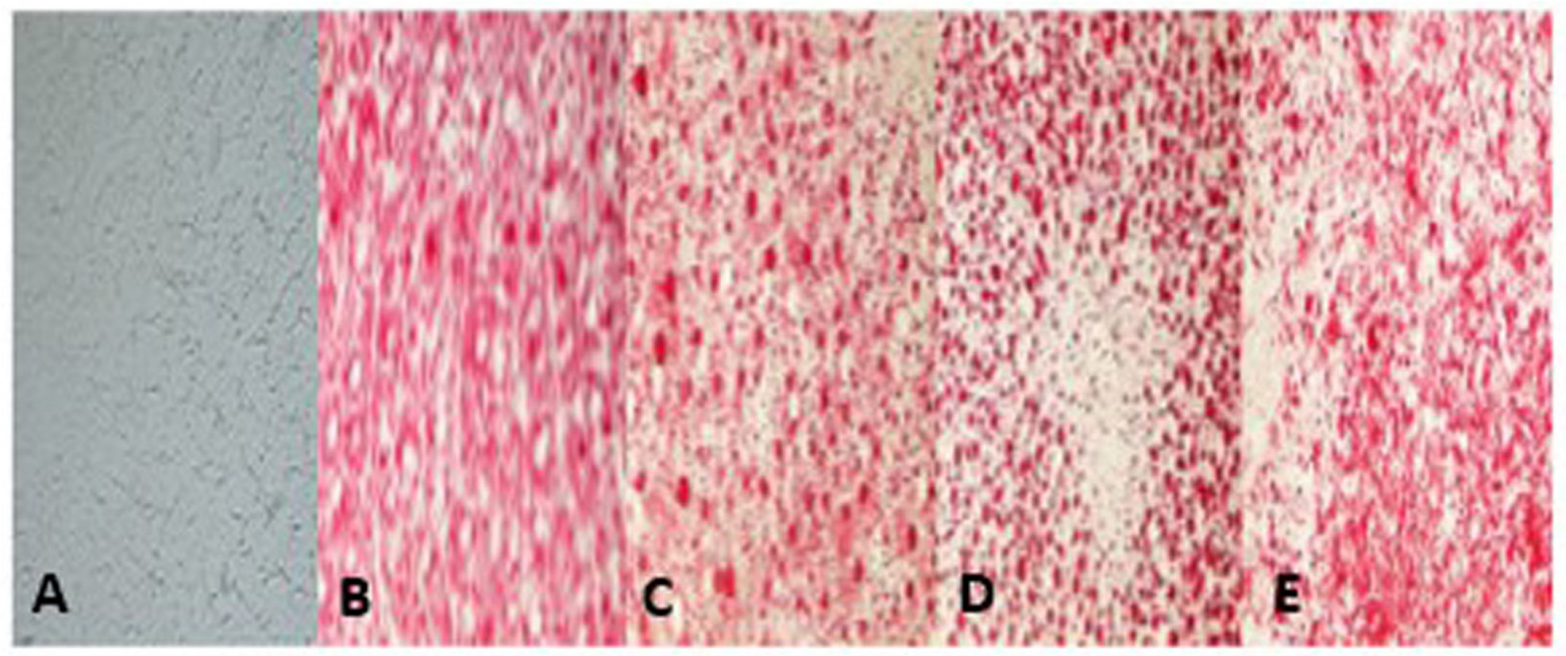
Results of frozen liver sections stained with Oil Red O in vivo (magnification, ×400). The red blots demonstrate lipid droplets in hepatocytes. Images from (A) chow-fed, (B) high-fat diet model, (C) GGQLD-low dose, (D) GGQLD-high dose and (E) resveratrol-treated rats. GGQLD, Gegen Qinlian decoction.

